# Cathodic reductive coupling of methyl cinnamate on boron-doped diamond electrodes and synthesis of new neolignan-type products

**DOI:** 10.3762/bjoc.11.21

**Published:** 2015-02-03

**Authors:** Taiki Kojima, Rika Obata, Tsuyoshi Saito, Yasuaki Einaga, Shigeru Nishiyama

**Affiliations:** 1 Department of Chemistry, Faculty of Science and Technology, Keio University, Hiyoshi 3-14-1 Kohoku-ku, Yokohama 223-8522, Japan; 2 Research and Education Center for Natural Sciences, Keio University, Hiyoshi 4-1-1 Kohoku-ku, Yokohama 223-8521, Japan; 3 International Institute for Integrative Sleep Medicine, University of Tsukuba, 1-1-1 Tennodai, Tsukuba, Ibaraki 305-8577, Japan; 4 Japan Science and Technology Agency (JST), CREST, Hiyoshi 3-14-1, Yokohama 223-8522, Japan

**Keywords:** boron-doped diamond (BDD) electrode, cathodic reduction, electrochemistry, electrosynthesis, neolignan

## Abstract

The electroreduction reaction of methyl cinnamate on a boron-doped diamond (BDD) electrode was investigated. The hydrodimer, dimethyl 3,4-diphenylhexanedioate (racemate/meso = 74:26), was obtained in 85% yield as the major product, along with small amounts of cyclic methyl 5-oxo-2,3-diphenylcyclopentane-1-carboxylate. Two new neolignan-type products were synthesized from the hydrodimer.

## Introduction

Numerous lignans and neolignans were found as secondary plant metabolites, and many of them are known to exhibit interesting biological activities [1]. Due to their plausible roles as defense substances of plants, lignans, neolignans, and their congeners are promising candidates for agricultural chemicals, and some of their antioxidant and/or anti-inflammatory properties may be utilized for biological research and as lead structures for chemotherapeutic agents. Despite consisting of two phenylpropane (C_6_–C_3_) fragments, the variety of carbon frameworks provides a huge library of lignans and neolignans [2–4]. As a result of their structural diversity, they have been targets of synthetic and biological investigations. Several synthetic approaches, including electrochemical oxidative coupling reactions mimicking biosynthetic pathways, were reported to construct the backbones of these molecules [5]. Recently, boron-doped diamond (BDD) electrodes have attracted a great deal of attention for their wide potential window against evolution of both hydrogen and oxygen and for their high stability which is derived from their diamond carbon structure [6]. Although anodic oxidation reactions mediated by BDD electrodes have been exploited in organic synthesis, there have been only few reports regarding their application in preparative-scale cathodic reduction of organic compounds [7].

**Figure 1:**
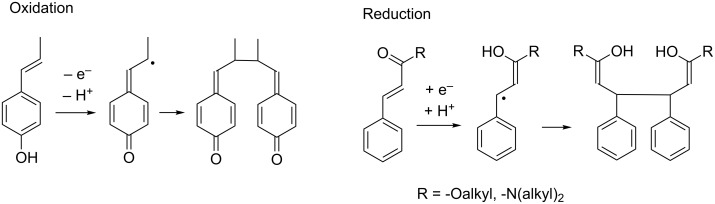
Expected coupling products from one-electron oxidation (left) and one-electron reduction (right) of C_6_–C_3_ compounds.

During our investigations of phenolic oxidation reactions using BDD electrodes, we observed the generation of solvent-derived methoxy radicals that conducted an oxidation process of the phenol substrate to the corresponding coupling product [8]. In our second investigation on the use of the BDD electrode in organic synthesis, the electrochemical reduction of methyl cinnamate (**1a**) was investigated to assess the applicability of BDD electrodes under cathodic reduction conditions, and to obtain new neolignan-type bioactive substances. As shown in Figure 1, the radical intermediate derived from phenylacrylate through a one-electron reduction (right) differs from that obtained by anodic oxidation of 4-hydroxyphenyl-1-propene (left). Therefore, the reductive dimerization of cinnamic acid derivatives was expected to provide access to unprecedented neolignan-type dimeric compounds.

## Results and Discussion

### Cathodic reduction on BDD electrode

The ester methyl cinnamate (**1a**) was electrolyzed under constant current electrolysis (CCE) conditions in a divided cell. Solvents used for the reactions played a significant role in providing the desired coupling (Table 1, entries 1–5). Thus, only acetonitrile (Table 1, entry 5) gave the desired coupling product (±)**-2** [9] in 4% yield, recovered educt **1a** and hydrolyzed product **1b**. The undesired hydrolysis could be depressed using a phosphate-buffered solution in the cathodic cell (pH 7, Table 1, entries 7–11), and finally the optimized conditions for the synthesis of **2** (85% yield, racemate/meso = 74:26) were acquired in the case of 2.5 F/mol current (Table 1, entry 11).

**Table 1:** Cathodic reduction of **1a** on a BDD electrode.

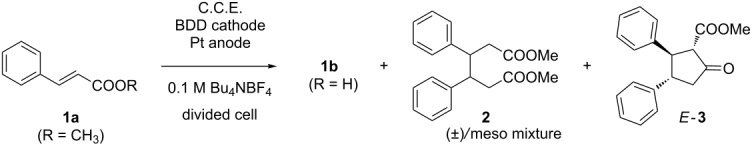								

Entry^a^	Solvent	Current density (mA/cm^2^)	Potential (V vs SCE)	F/mol	Yield (%)^b^			

					**1a**	**1b**	**2** [(±)/meso)]^c^	**3**^d^

1	DMSO	0.21	−2.08 to −1.93	1	32	51	0	0
2	DMF	0.50	−1.96 to −1.86	1	43	43	0	0
3	TFE^e^	0.53	−2.00 to −1.85	1	100	0	0	0
4	MeOH	1.29	−2.08 to −1.84	1	74	12	0	0
5	MeCN	1.29	−2.00 to −1.88	1	42	46	4 (100/0)	0
6	MeCN^f^	1.29	−2.21 to −1.98	1	10	23	19 (79/21)	3
7	MeCN^g^	1.29	−2.07 to −1.89	1	23	13	33 (85/15)	5
8	MeCN^h^	1.29	−1.91 to −1.83	1	45	0	44 (73/27)	3
9	MeCN^h^	1.29	−2.02 to −1.84	1.5	26	0	67 (73/27)	5
10	MeCN^h^	1.29	−2.00 to −1.82	2.0	15	0	70 (73/27)	5
11	MeCN^h^	1.29	−2.12 to −1.93	2.5	1	0	85 (74/26)	4

^a^Upon using undivided cell systems, the reaction proceeded slower than in the divided cell cases, and lower selectivity of **2** and **3** was observed. ^b^Isolated yields. ^c^The ratio of (±) and meso forms was determined by ^1^H NMR spectroscopy. ^d^Enantiomeric mixture. ^e^2,2,2-Trifluoroethanol. ^f^Containing 0.07 M pH 6.0 phosphate buffer. ^g^Containing 0.07 M pH 7.0 phosphate buffer. ^h^Containing 0.33 M pH 7.0 phosphate buffer.

To check for a different behavior of the BDD electrode, several electrode materials, including glassy carbon (GC), platinum (Pt), and magnesium (Mg), were examined as cathodes under the optimized electrolytic conditions (Table 1, entry 11). Hydrogen evolution at the electrode was recognized when Pt and Mg electrodes were used, and the educt **1a** was recovered in high yield. The GC electrode provided the coupling product **2** (34%, racemate/meso = 74:26) and *E*-**3** (25%), along with 41% of **1a**. Similar cathodic reductions of cinnamate derivatives were carried out using Hg [10–11], Cu [12–13], Pb [13–14], Zn [13], Sn [13], and Ag [13], and the major products were the cyclic products (type **3**) through Diekmann-type cyclization, whereas the hydrodimer **2** was the predominantly produced product in the present BDD electrode mediated reduction. Despite a different product ratio, the GC electrode gave similar reaction products to that of the BDD electrode.

### Synthesis of new neolignans

**Scheme 1:**
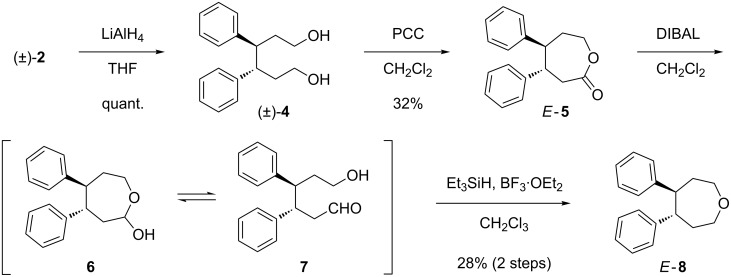
Chemical conversion of (±)-**2** into *E*-**5** and *E*-**8**.

As shown in Scheme 1, after separation of the diastereomeric mixture, (±)-**2** was submitted to the chemical conversion into the new neolignan-type derivatives *E*-**5** and *E*-**8**. Thus, reduction of (±)-**2** with LiAlH_4_ gave the alcohol (±)-**4** [15] in quantitative yield, which on oxidation with PCC [16] gave the lactone *E*-**5** in 32% yield. Selective DIBAL reduction of *E*-**5** gave an inseparable mixture of **6** and **7**, which were identified by ^1^H NMR spectroscopy. Subsequent treatment of the mixture with Et_3_SiH in the presence of BF_3_·OEt_2_ finally gave *E*-**8**.

## Conclusion

The cathodic reduction of **1a** using BDD electrode predominantly gave the dimeric product **2** in 85% yield. A remarkable solvent effect of MeCN was observed for this dimerization reaction, while stereoselectivity was unaffected among the conditions tested and the racemic form was predominant over the meso form in all cases. Electrochemically prepared (±)*-***2** was further converted into *E*-**5** and *E*-**8** as novel unprecedented neolignan-type derivatives. These results provide an example for an electroorganic synthesis using cathodic reductive coupling on a boron-doped diamond electrode.

## Supporting Information

File 1 Instrumental setup, general procedure for the electrochemical reaction and physical and spectroscopic data for (±)*-***2**, meso-**2**, *E*-**5**, and *E*-**8**.
