# Supplementary material for: Cathodic reductive coupling of methyl cinnamate on boron-doped diamond electrodes and synthesis of new neolignan-type products
Source: Beilstein J Org Chem. 2015 Feb 3;11:200–3. doi: 10.3762/bjoc.11.21 (PMC4361996; doi:10.3762/bjoc.11.21)
Supplement: File 1 — Instrumental setup, general procedure for the electrochemical reaction and physical and spectroscopic data for (±)-2, meso-2, E-5, and E-8. [file Beilstein_J_Org_Chem-11-200-s001.pdf]

Supporting Information
for
Cathodic reductive coupling of methyl cinnamate on boron-doped diamond electrodes and synthesis of new neolignan-type products

Taiki Kojima[1], Rika Obata[2], Tsuyoshi Saito[3,4], Yasuaki Einaga*[1,4], and Shigeru Nishiyama*[1,4]

Address: [1]Department of Chemistry, Faculty of Science and Technology, Keio University, Hiyoshi 3-14-1 Kohoku-ku, Yokohama 223-8522, Japan, [2]Research and Education Center for Natural Sciences, Keio University, Hiyoshi 4-1-1 Kohoku-ku, Yokohama 223-8521, Japan, [3]International Institute for Integrative Sleep Medicine, University of Tsukuba, 1-1-1 Tennodai, Tsukuba, Ibaraki 305-8577, Japan and [4]Japan Science and Technology Agency (JST), CREST, Hiyoshi 3-14-1, Yokohama 223-8522, Japan

Email: Yasuaki Einaga - einaga@chem.keio.ac.jp; Shigeru Nishiyama - nisiyama@chem.keio.ac.jp

*Corresponding author

Instrumental setup, general procedure for the electrochemical reaction and physical and spectroscopic data for (±)-2, meso-2, *E*-5, and *E*-8

1. Electrolysis Apparatus

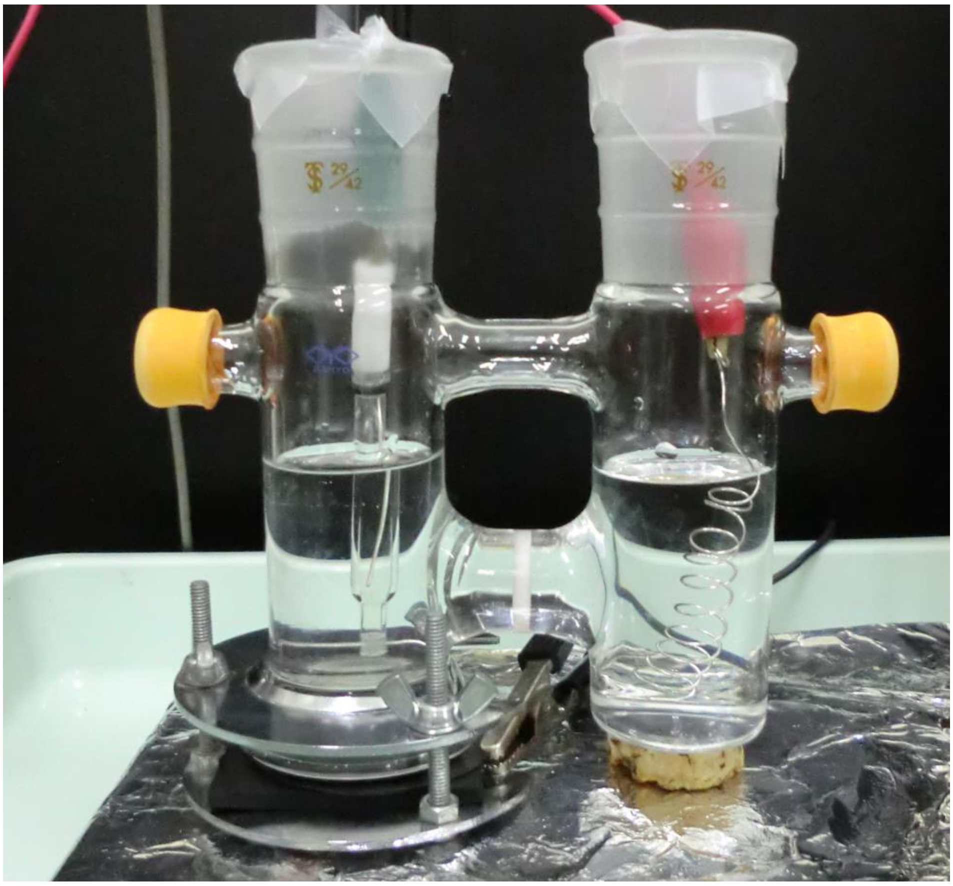

BDD electrode

2. General electrochemical procedure.

Preparation of BDD plate (7 cm^2) was indicated in Supporting Information of [1]. Electroreduction was carried out at room temperature with BDD plate as a cathode, platinum wire as an anode, and saturated calomel electrode as a reference electrode under CCE conditions in a divided cell. Cathode and anode parts were separated with a glass filter. Substrate concentration was 40 mM in solvents (40 mL) containing 0.1 M nBu_4NBF_4. The reaction was terminated, when current reached to the indicated amount.

3. Data of (±)-**2**, meso-**2**, *E*-**5**, and *E*-**8**.

Dimethyl (3*S**,4*S**)-3,4-diphenylhexanedioate [(±)-**2**]: mp 74-76 °C (hexane/EtOAc), 74.5–75°C [2]; IR (film) 1737 cm^{-1}; ^{1}H NMR ($CDCl_3$) δ 7,20-7.13 (m, 6H), 6.87 (dd, 4H, *J* = 7.6, 1.6 Hz), 3.55 (s, 6H), 3.53-3.47 (ddd, 2H, *J* = 10.8, 8.5, 6.1 Hz), 2.75 (dd, 2H, *J* = 15.7, 6.1 Hz), 2.63 (dd, 2H, *J* = 15.7, 8.5 Hz); ^{13}C NMR ($CDCl_3$) δ 172.6, 140.1, 128.9, 128.0, 126.9, 51.8, 46.4, 37.7. HRMS (ESI-MS): calcd. for $C_{20}H_{23}O_4$ 327.1596 $(M+H)^+$, found *m/z* 327.1583.

Dimethyl (3*R**,4*S**)-3,4-diphenylhexanedioate (meso-**2)**: mp 177-178 °C (hexane/EtOAc), 175–176 °C [2]; IR (film) 1726 cm^{-1}; ^{1}H NMR ($CDCl_3$) δ 7.35-7.22 (m, 10H), 3.36 (s, 6H), 3.32-3.30 (ddd, 2H, *J* = 11.0, 8.1, 4.0 Hz), 2.46 (dd, 2 H, *J* = 15.7, 8.1 Hz), 2.37 (dd, 2 H, *J* = 15.7, 4.0 Hz); ^{13}C NMR ($CDCl_3$) δ 172.5, 141.9, 128.9, 128.3, 127.3, 51.5, 47.9, 39.9. HRMS (ESI-MS): calcd. for $C_{20}H_{23}O_4$ 327.1596 $(M+H)^+$, found *m/z* 327.1611.

(4*S**,5*S**)-4,5-Diphenyloxepan-2-one (*E*-**5**): mp 173–175 °C (hexane/EtOAc); IR (film) 1720 cm^{-1}; ^{1}H NMR ($CDCl_3$) δ 7.13–7.02 (m, 6H), 6.92–6.90 (m, 4H), 4.54 (m, 2H), 3.25 (dd, 1H, *J* = 13.0, 11.7 Hz), 3.12 (ddd, 1H, *J* = 11.7, 11.7, 12.6 Hz), 3.04 (ddd, 1H, *J* = 11.7, 8.5, 7.2 Hz), 2.87 (dd, 1H, *J* = 13.0, 12.6 Hz), 2.26–2.21 (m, 2H); ^{13}C NMR ($CDCl_3$) δ 174.6, 143.7, 143.5, 128.5, 127.5, 127.1, 126.7, 126.6, 68.3, 54.0, 46.6, 41.3, 37.6. HRMS (ESI-MS): calcd. for $C_{18}H_{19}O_2$ 267.1385 $(M+H)^+$, found *m/z* 267.1382.

(4*S**,5*S**)-4,5-Diphenyloxepane (*E*-**8**): mp 95–97 °C (hexane/EtOAc); IR (film) 1125 cm^{-1}; ^{1}H NMR ($CDCl_3$) δ 7.07 (t, 4H, *J* = 7.2Hz), 6.99 (d, 2H, *J* = 7.2Hz), 6.94 (ddd, 4H, *J* = 7.2,1.1 Hz), 4.00 (ddd, 1H, *J* = 12.1, 4.0, 4.0 Hz), 3.88 (ddd, 1H, *J* = 9.4, 9.4, 2.7 Hz), 3.03 (ddd, 2H, *J* = 7.4, 5.3, 2.9 Hz), 2.32–2.22 (m, 2H), 2.03 (ddd, 2H, *J* = 3.1, 2.9, 2.4 Hz); ^{13}C NMR ($CDCl_3$) δ 146.4, 128.1, 127.6, 125.7, 67.5, 51.9, 38.6. HRMS (ESI-MS): calcd. for $C_{18}H_{21}O$ 253.1592 $(M+H)^+$, found *m/z* 253.1603.

4. References

[1] Sumi, T.; Saitoh, T.; Natsui, K.; Yamamoto, T.; Atobe, M.; Einaga, Y.; Nishiyama, S. *Angew. Chem., Int. Ed.* **2012,** *51,* 5443–5446.
[2] Curtin, D. Y.; Dayagi, S. *Can. J. Chem*., **1964**, *42*, 867–877.
